# Intraspecific geographic variation in rod and cone visual pigment sensitivity of a parrot, *Platycercus elegans*

**DOI:** 10.1038/srep41445

**Published:** 2017-01-27

**Authors:** Ben Knott, Mathew L. Berg, Raoul F. H. Ribot, John A. Endler, Andrew T. D. Bennett

**Affiliations:** 1 Deakin University, Geelong, Australia, School of Life and Environmental Sciences, Centre for Integrative Ecology, Locked Bag 20000, Geelong, VIC 3220, Australia

## Abstract

Variation in wavelength sensitivity among subspecies is unknown among vertebrates. The parrot *Platycercus elegans* has extreme plumage variation between subspecies ranging from pale yellow to crimson which, with differences in background colour and light environment between subspecies, makes it a good candidate for the evolution of within-species differences in vision. We report differences in visual pigments between populations of *P. elegans* from two subspecies, providing the first known support for population and subspecies variation in visual pigments within a vertebrate species; it is also the first instance of intraspecific variation in rod sensitivity within any vertebrate species. Differences in wavelength sensitivity of rods and cones corresponded to geographic differences in plumage colour. Between study populations, visual pigments varied but not oil droplets. Adaptive functions for the visual pigment differences are untested but they could cause divergence in behaviours associated with colour as well as in dim light, and provide insights into the role of senses in divergence and speciation.

The rod and cone photoreceptors in the retina of the vertebrate eye are the fundamental basis for nocturnal and diurnal vision respectively, and the sensitivities of these photoreceptors have evolved to suit the visual needs of individuals in each species^1^. Vision within a species is thought to be broadly conserved between individuals and across populations within a species, including in birds^2^,^3^. However, most studies assessing adaptations in vision compare species, rather than separate populations within a species that occupy different niches^4^,^5^,^6^,^7^. Intraspecific variation in photoreceptor sensitivity is known from only a few vertebrate species, namely some teleost fish^7^,^8^,^9^ and primates (e.g. some New World monkeys^10^,^11^ and humans^12^,^13^). In these cases, intraspecific variation arises from within-population differences caused by differences between individuals in retinal photopigment alleles and/or their expression^8^,^11^,^13^. To date, intraspecific variation in vision has not been found between geographically separate populations of any species, nor within any bird species, nor in the rods of any vertebrate species.

The parrot *Platycercus elegans* (Gmelin 1788, Aves; Psittaciformes) shows extreme variation in plumage colouration between subspecies, ranging from deep crimson (in *P. e. elegans*) to pale yellow (in *P. e. flaveolus*)^14^. *P. e. elegans* occupies mesic wooded and forest habitats, and *P. e. flaveolus* more open riparian habitats^14^. *Platycercus elegans* is perhaps the most colour variable of the *ca.* 350 species of parrot worldwide and, based on the plumage variation, Cain^15^ considered the species an example of a circular overlapping or ‘ring’ species^16^,^17^, of which there are few worldwide. Recent studies of *P. elegans* focusing on population structure^18^, vocalisations^19^,^20^,^21^,^22^, olfaction^23^ and viral infection^24^, however, have revealed a more complex phylogeography than proposed in the simple ‘ideal’ ring species model proposed by Mayr^17^. Of particular interest are the selective pressures driving and maintaining the evolution of the plumage colour variation in *P. elegans*, many of which remain enigmatic^25^. Sensory drive theories^26^,^27^, in combination with predictions arising from the known differences in the subspecies’ light environments and background colouration^1^,^27^, make it is possible that consistent intraspecific differences in vision may have evolved in the different subspecies in *P. elegans* living in different habitats. Indeed, the molecular sequences of the retinal opsins of *P. e. adelaidae*, a subspecies with colouration intermediate to *P. e. elegans* and *P. e. flaveolus* reveal sequence extensions and splicing events previously unknown in any vertebrate^28^. Using the two most divergent subspecies of the complex (*P. e. elegans* and *P. e. flaveolus*) we compared their rods and all known cone types for spectral sensitivity differences; our approach allowed us to partition differences due to visual pigments, or the oil droplets that overlie visual pigments in birds.

## Results

**Figure 1:**
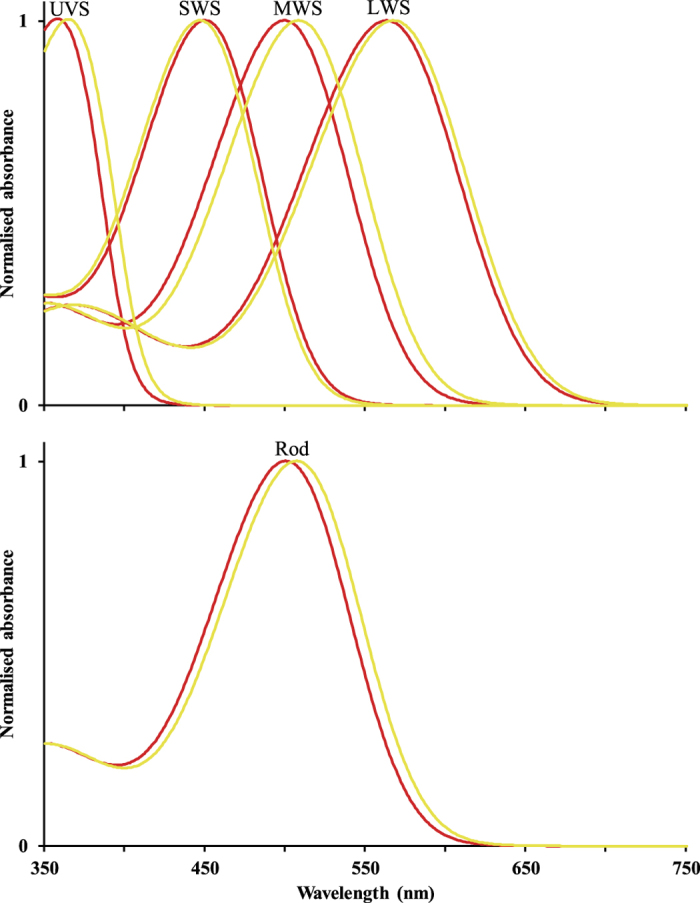
Visual pigment templates^59^ for the mean λmax from all individual cells for *P. e. elegans* (red) and *P. e. flaveolus* (yellow) shown separately for each visual pigment type. Top panel: (L-R) UVS, SWS, MWS, LWS. Bottom panel: Rod.

**Figure 2:**
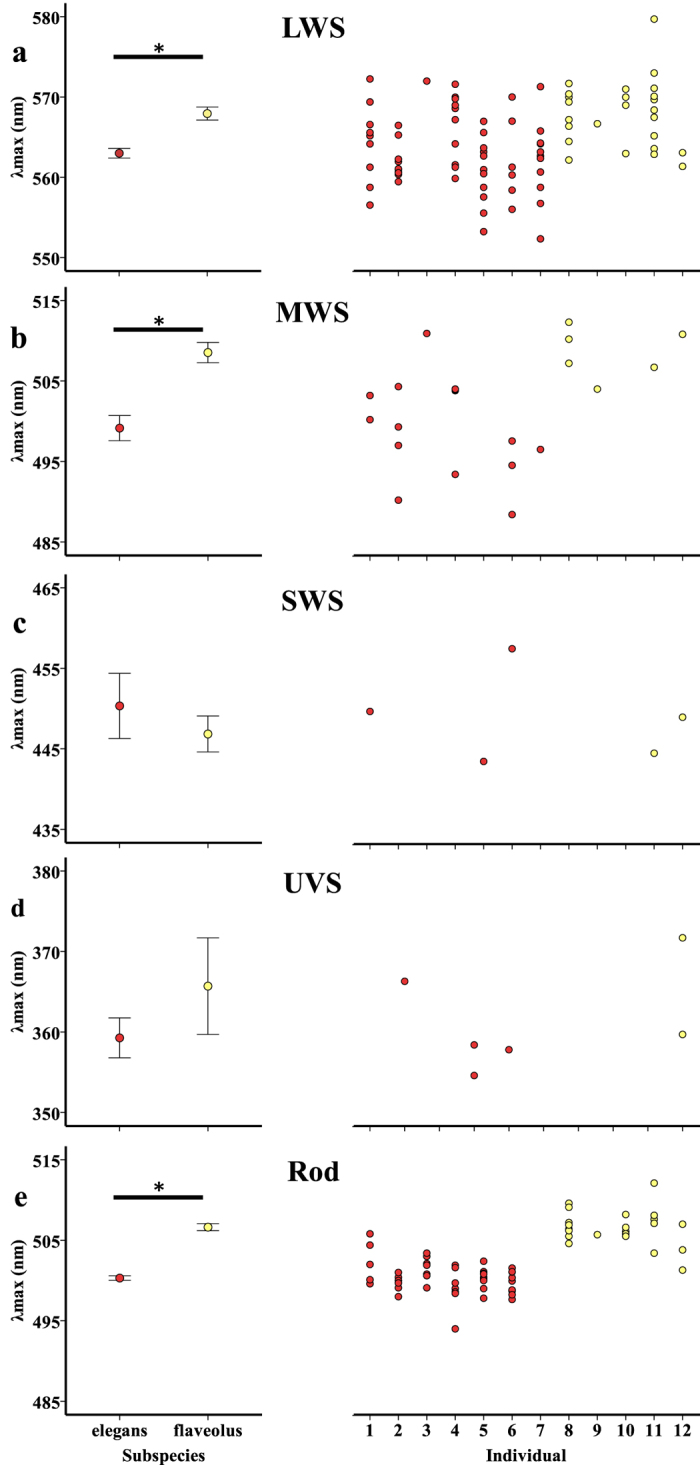
Left column: Mean visual pigment λ_max_ for *P. e. elegans* and *P. e. flaveolus* for each visual pigment type. Error bars represent ± 1 standard error. Significant results are indicated by a black line and asterisk. Right column: Spread of all individual photoreceptor cell λ_max_ for each individual bird, grouped by subspecies: Individuals 1–7: *P. e. elegans*; Individuals 8–12: *P. e. flaveolus*. Rows: a: LWS; b: MWS; c: SWS; d: UVS; e: Rod.

Microspectrophotometry (MSP) revealed all the expected parrot visual pigment types^28^,^29^,^30^ in both the *P. e. elegans* and the *P. e. flaveolus* populations (Table 1; Fig. 1). For the rod, longwave sensitive (LWS), and mediumwave sensitive (MWS) cone visual pigments, we found significant differences in the wavelength of maximum sensitivity (λ_max_) between the two study populations (Table 1; Fig. 2a,b,e), with those from the *P. e. flaveolus* population at consistently longer wavelengths than visual pigments from the *P. e. elegans* population (Table 1; Fig. 2a,b,e). There were non-significant differences between sub-species in λ_max_ of the shortwave sensitive (SWS) and ultraviolet sensitive (UVS) cone visual pigments (Table 1; Fig. 2c,d). As expected from numerous terrestrial vertebrate studies, photoreceptors containing SWS and UVS visual pigments were infrequent. The intraclass correlation coefficients (ICC) were 0.30, 0.55, and 0.75 for LWS, MWS and rod visual pigments respectively. These indicate that 30%, 55%, and 75% of variance was associated with differences between individuals for LWS, MWS, and rod visual pigments respectively. The ICCs were not calculated for SWS and UVS because there were too few repeated measurements across individuals. Data were normally distributed and homoscedastic. These ICC values are within the range reported for earlier MSP work on bird eyes, both in cone outer segments and in oil droplets^31^. Because we have sampled from a single population of each subspecies we cannot say whether this is a population or subspecies difference, but the large ICC indicate that the intraspecific differences are real.

**Figure 3:**
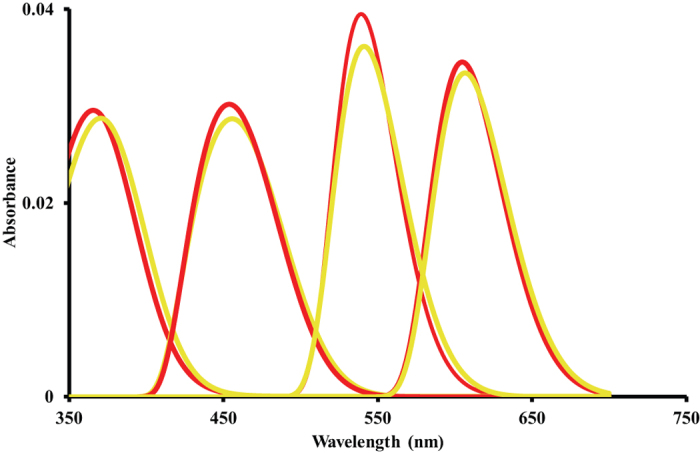
Eye models for *P. e. elegans* (red) and *P. e. flaveolus* (yellow), showing the predicted sensitivity curves for the four single cone photoreceptors based on the visual pigment and oil droplet microspectrophotometry data collected in this study.

MSP revealed all expected parrot retinal oil droplets types (Table 2). No significant differences in the cut-off wavelength (λ_cut_)^32^ were found between the populations for any oil droplet types (Table 2). Predicted sensitivity curves for single cones, incorporating visual pigment sensitivity, oil droplet absorbance and cone ratios are shown in Fig. 3.

## Discussion

We report the first case in a vertebrate of intraspecific differences in spectral sensitivity corresponding to population and geographic differences. These differences occurred in both cones and rods, and the rod difference is the first case of intraspecific variation in rods in any vertebrate. In the cones, the differences in spectral sensitivity were due to differences in visual pigments rather than oil droplets. Where there were significant differences in visual pigments (LWS, MWS, rods) the population exhibiting plumage dominated by longer wavelength reflectance (*P. e. elegans*) had visual pigments sensitive to shorter wavelengths. Consequently, our results offer the first evidence in a bird that intraspecific geographic variation in photoreceptor tuning relates to integument colouration, and provides the first example of intraspecific variation in avian colour vision.

We show that significant differences exist between *P. e. elegans* and *P. e. flaveolus* in the peak absorbance of the visual pigments contained in both rod, LWS and MWS cone photoreceptors. This confirms an *a priori* prediction that we tested earlier in budgerigar colour morphs^31^ in which we found no differences in oil droplets between colour morphs. However, the budgerigar colour morphs tested were produced by captive breeding and do not occur in the wild, where there is a single wild-type morph^33^. Here, using wild-caught individuals of a species, *P. elegans*, with naturally occurring extreme intraspecific plumage variation, we found consistent differences, and in several visual pigments. The selective advantages of these differences in *P. elegans* are not yet clear, but may relate to differences in habitat background coloration and light environment between *P. elegans* subspecies, combined with sensory drive theories^34^.

We did not find any differences between the two populations that we studied in the absorbance properties of oil droplet types (i.e. R-, Y-, C- and P-types). As in the great majority of bird species, and in our study reported here, the R-, Y-, C- and T-type oil droplets of single cones sit in the optical paths of the visual pigments LWS, MWS, SWS and VS/UVS, respectively. The T-type droplet has no detectable absorbance in birds, and the P-type droplet is found exclusively in the double cones^2^,^35^. Work on developing chicks^36^ found 6 weeks of manipulated ambient light was required for changes in oil droplet absorbance. Our previous research on adult *P. elegans*^37^ revealed that medium term (*ca*. 90 days) dietary manipulations of carotenoids and food availability did not affect oil droplets of single cones, and only affected the P-type droplets of double cones that apparently have no role on colour vision^38^,^39^. In the work reported here on adult *P. elegans*, the time birds were held in captivity prior to MSP was around 25 days. Consequently, there is no reason to conclude that being held in captivity after wild-capture eliminated naturally occurring differences between populations in oil droplet absorbance.

Differential expression of polymorphic alleles of the genes coding for the protein opsins that comprise the visual pigments are the key mechanisms underlying intraspecific variation in visual pigments observed in taxa other than birds. For example, in guppies, individuals within a population express a subset of four LWS and two SWS cone opsin alleles^40^,^41^. In New World monkeys, multiple alleles of a single sex-linked locus on the X-chromosome leads to dichromatic and trichromatic individuals within a population^42^. Environment^43^ and age^44^ can also influence expression of different alleles in primates. LWS visual pigment polymorphisms are sex linked in humans, and lead to red-green colour blindness in about 8% of men^45^, although evolutionary causes of human LWS polymorphisms remain contested^46^.

Our data on *P. elegans* alludes to subspecies differences in vision, but does not indicate the evolutionary origins of these differences. However, it is plausible that habitat differences could have been a contributory factor in the divergence of the plumage colouration in the *P. elegans* complex^27^. *P. e. elegans* is found in mesic wooded and forest habitats whereas *P. e. flaveolus* is found in more open riparian habitats dominated by *Eucalyptus camaldulensis*. In forests, ambient light contains reduced long wavelengths and appears green to yellow-green to the human eye when compared to ambient light in open habitats^47^. As such, one might expect animals living in forest shade to show shifts to shorter wavelengths in their visual pigment sensitivity in response to the ambient light, and this is the shift we observe in the LWS and MWS cones, and the rods of *P. e. elegans* when compared to *P. e. flaveolus*. The differences in λ_max_ observed between *P. e. elegans* and *P. e. flaveolus* in LWS and MWS (Table 1) are close to, or larger than, the differences discovered between polymorphisms of the LWS visual pigment in humans, a difference which has been demonstrated many times to affect colour perception in colour matching experiments^48^. The ecological and behavioural factors that underlie the tuning of visual pigments in *P. elegans*, such as possible roles in mate choice, foraging, or navigation, remain to be determined. The significant differences in the λ_max_ of the two cone visual pigments may lead to differences between our populations in their colour perception. Modelling to predict consequences for visual discrimination in *P. elegans* will need to consider filtering effects of the oil droplets (Fig. 3) plus the role of habitat light environments and background coloration which are likely to vary between habitats, along with the known plumage reflectances between the two subspecies. If the different visual pigments in the two subspecies did have negligible effects on visual discrimination, then the visual pigment difference would be likely due to genetic drift. However, even if the different visual pigments only have small effects on discrimination, or only in certain contexts, given enough time in the right environment they could still have selective effects, for as pointed out by Fisher^49^, even small differences in selective advantage will cause significant evolution.

Differences between populations in the λ_max_ of the rod photoreceptors used for dim light vision are intriguing. The causes of this shift are difficult to assess given there is little understanding of the factors underlying the spectral tuning of rod photoreceptors^2^,^50^. During twilight, spectral irradiance of skylight is of greatest intensity between 450–500 nm^47^,^51^, and a rod λ_max_ of approximately 500 nm, as observed in *P. e. elegans*, would be reasonably well adapted to vision in these conditions^2^. At night longer wavelengths of light become relatively more abundant, and a 500 nm λ_max_ would no longer be optimal^51^. Potentially, the longer λ_max_ of 506 nm observed in the *P. e. flaveolus* population could indicate a behavioural change in this subspecies to greater activity at dawn and dusk, where such a sensitivity shift would collect more light from leaves^47^ though any differences in activity between subspecies remains untested. However, even in nocturnal birds, rod λ_max_ is usually located at approximately 500 nm^52^, even though longer wavelengths are more abundant^51^, so the causes of the intraspecific variation in the rods remain unclear. While few conclusions can be drawn from these data alone, the reported differences in rod λ_max_ may be indicative of a behavioural shift in one subspecies, or an adaptation to maintain similar behaviours in different light environments. An alternative explanation could be that the observed λ_max_ shift is a consequence of selection for a different aspect of opsin performance. For example, the λ_max_ of avian rod visual pigments has been suggested to be a trade-off between optimal spectral sensitivity for dim light conditions, and resistance to ‘dark noise’ i.e. when an opsin is randomly activated by heat instead of light^53^. Potentially, the known differences in habitat between subspecies (*flaveolus* habitat is riverine) may mean the vision of one subspecies is more affected by dark noise than the other, so the opsins may have adapted to be more resistant. However, the general lack of knowledge of the factors influencing rod spectral sensitivity means any assessment of the differences in rod λ_max_ are highly speculative.

Our earlier sequencing of retinal opsins of *P. elegans*^28^ revealed the full complement expected in birds^3^, namely four cone opsins (SWS1, SWS2, RH2 and LWS) and a single rod opsin, RH1. Those data^28^ came from another subspecies, *P. e. adelaidae* so their relevance to *P. e. elegans* and *P. e. flaveolus* may be limited, but they are the best data available on *P. elegans*. Consequently, we suggest it is likely *P. e. elegans* and *P. e. flaveolus* both only have SWS1, SWS2, RH2, LWS and RH1. We suggest the geographic differences in visual pigment tuning that we report here are most likely caused by divergence over time of the individual opsin genes for each visual pigment in each population, and not through differential expression of a subset of alleles. Potentially, the visual pigment sensitivity differences reported here may have arisen simply through isolation of the two populations which we sampled. However, previous analyses of the population genetics of the *P. elegans* complex suggest there is ongoing gene flow, little reproduction isolation between *P. e. elegans* and *P. e. flaveolus* subspecies, and the subspecies are not geographically isolated^18^,^22^. Given the correlation between an animal’s vision and its visual environment^1^, we suggest the divergence of visual pigment sensitivity between the *P. e. elegans* and *P. e. flaveolus* is unlikely simply through reproductive isolation, though whether or not the divergence is a consequence of differences in the visual environment remains unknown. As discussed for the rod visual pigment, the differences in λ_max_ of the cones could also be a side effect of selection on a different aspect of visual pigment biochemistry, such as their resistance to dark noise. However, we suggest that such a change is likely to be generally advantageous to both subspecies and so we would expect the trait in both subspecies.

## Conclusions

We reveal hitherto unknown diversity in the visual pigments of birds which, to date, had been regarded as highly conserved across taxa^2^,^3^. Additional MSP investigation to assess the extent of the divergence within and between the ranges of each subspecies will enable assessment of relationships between visual pigment sensitivity, visual environment, and the plumage colouration of the various subspecies of *P. elegans*. If a relationship is established, this could elucidate the role of the senses in speciation and the generation of biodiversity, because differences in perception of sexual signals can lead to further divergence of the signals, which can feedback to further divergence in vision^27^,^33^.

Taken together, our findings suggest that within-species differences in the physiology of vision may be more widespread than hitherto considered. As systematic investigation of within species differences in vertebrate vision have been so rarely attempted, our work suggests that testing and modelling such differences may be a fruitful area of future research. Classic examples of evolution from mimicry, to warning coloration and crypsis are usually tested on organisms such as insects which signal their coloration to birds as predators^26^,^54^ so improved understanding of factors tuning the avian eye – the most complex visual system of any vertebrate – will be of substantial value. Our findings have implications which range from better understanding of the role sensory processes play in divergence and speciation, to improved understanding of the evolution of the vertebrate eye and factors tuning its photoreceptors.

## Methods

### Subjects

Birds were caught from the wild at two populations, which represented two subspecies of *P. elegans*. Seven *P. e. elegans* were caught in Bellbrae, Victoria, Australia (S38°20′S, E144°16′S), and five *P. e. flaveolus* were caught near Mildura, Victoria, Australia (S34°25S, E142°18S). Birds were transported to Deakin University, Geelong, Australia and held for about 3 weeks (mean 25 days +/−27 SD) in outdoor flight cages on *ad libitum* parrot seed mix, fresh fruit and water, until retinal examination via MSP. For this, subjects were dark adapted for at least one hour before their humane killing.

### Microspectrophotometry

Eyes were enucleated, and retinal tissue samples were prepared for MSP under infrared light using methods reported previously^28^,^55^. MSP was performed using a computer-controlled single beam device at Deakin University, Geelong, Australia. Measuring beams were aligned to pass transversely through the photoreceptor outer segment and run in 2 nm intervals from 750 nm to 350 nm, then back from 351 nm to 749 nm. After each measurement, the pigment was bleached with white light and the outer segment was rescanned to confirm the post-bleaching disappearance of the pigment and appearance of short-wavelength absorbing photoproducts. Oil droplet absorbance was measured using the same MSP scanning protocol, though with no post-scan bleaching.

### λ_max_ and λ_cut_ calculation

Prior to λ_max_ calculation, all MSP records for each individual were randomly assigned to numbered files, such that the researcher calculating the λ_max_ (BK) was blind to the population origin of any particular set of measurements. λ_max_ was calculated from individual cell records for each pigment type using a standardised computer program^29^,^56^: briefly, absorbance values at pairs of adjacent wavelengths were averaged to obtain a mean curve from outward and return scans. λ_max_ were calculated by fitting the 20 absorbance values on the long wavelength limb to a standard template curve^57^ to give an average λ_max_. This analysis effectively finds the spectral location of the standard curve that would produce the per cent absorbance values being considered^28^,^29^,^56^. For data selection purposes, a second estimate of λ_max_ was obtained by fitting each of 50 absorbance points, centred on the peak of the averaged absorbance curve to the template curve, and averaging the results. Following λ_max_ calculation, each cell record was subject to rigid selection criteria^29^. For LWS, MWS and rod pigments, spectra required a transverse density greater than 0.01, a standard deviation from the right hand limb of less than 12 nm, and a difference between the two estimation methods of less than 6 nm. For SWS and UVS, the criteria were relaxed due to the rarity of these cell types^2^, and any cells showing convincing evidence of post-measurement bleaching by white light were retained.

Oil droplet λ_cut_ were calculated using the method of Lipetz^32^. Briefly, a tangent is fitted to the droplet absorbance spectra at 50% maximum absorbance, and extrapolated. The wavelength at which the extrapolated tangent crosses the maximum measured absorbance of the spectra is the calculated λ_cut_.

### Geographic λ_max_ and λ_cut_ comparison

For each population, records satisfying the selection criteria were analysed using SPSS 21 (SPSS Inc.). We analysed λ_max_ data for each pigment type (LWS, MWS, SWS, UVS, rod) using linear mixed models (LMMs), with restricted maximum likelihood estimation. These models included a fixed effect for population/subspecies of origin (*P. e. elegans* or *P. e. flaveolus*), and a random intercept comprising bird ID. The random effect was included to account for non-independence of visual pigments measured from each bird. We report estimates of fixed effects and pairwise comparisons based on estimated marginal means resulting from these models. Significance of fixed effects was assessed using type-III sums of squares. λ_cut_ data were analysed using the same methods. The intraclass correlation coefficient (ICC), which is the ratio of variance between individuals to the total variance^58^, was calculated for each visual pigment type using type using intercepts-only mixed models.

For visualisation of the visual pigment absorbance spectra, visual pigments templates were produced using the mean values generated by the above analyses, using the methods of Govardovskii *et al*.^59^.

### Ethics statement

All work was approved by Deakin University Animal Ethics Committee (approval no. G08–2012), and conducted under permit from the Victorian Department of Environment & Primary Industries (wildlife research permit no. 10006284). All methods were performed in accordance with the relevant guidelines and regulations.

## Additional Information

**How to cite this article**: Knott, B. *et al*. Intraspecific geographic variation in rod and cone visual pigment sensitivity of a parrot *Platycercus elegans. Sci. Rep.* **7**, 41445; doi: 10.1038/srep41445 (2017).

**Publisher's note:** Springer Nature remains neutral with regard to jurisdictional claims in published maps and institutional affiliations.

## Figures and Tables

**Table 1:** Mean λ_max_ and statistics for the comparison of visual pigment sensitivity in *P. e. elegans* and *P. e. flaveolus*.

Visual pigment	*P. e. elegans*		*P. e. flaveolus*		Numerator df	Denominator df	F	P
	No. cells	Mean λ_max_ ± SE	No. cells	Mean λ_max_ ± SE				
**LWS**	**59**	**563.2** **±** **0.8**	**26**	**567.6** **±** **1.1**	**1**	**8**	**10.1**	**0.013**
**MWS**	**15**	**499.5** **±** **1.7**	**6**	**508.3** **±** **2.5**	**1**	**7**	**8.5**	**0.023**
SWS	3	450.3 ± 3.5	2	446.8 ± 4.3	1	3	0.4	0.571
UVS	4	359.3 ± 3.0	2	365.7 ± 4.3	1	4	1.5	0.287
**Rod**	**46**	**500.4** **±** **0.4**	**24**	**506.4** **±** **0.6**	**1**	**8**	**57.9**	**<0.001**

Significant results are shown in bold. No. cells indicates the number of cells passing the selection criteria.

**Table 2:** Mean λ_cut_ and statistics for the comparison of oil droplet absorbance in *P. e. elegans* and *P. e. flaveolus*.

Droplet	P. e. elegans		P. e. flaveolus		Numerator df	Denominator df	F	P
	No. cells	Mean λ_cut_ ± SE	No. cells	Mean λ_cut_ ± SE				
R-type	18	569.1 ± 0.4	9	569.3 ± 0.6	1	25	0.05	0.833
Y-type	40	408.2 ± 0.7	16	506.2 ± 1.1	1	54	2.4	0.128
C-type	5	406.1 ± 1.6	3	407.9 ± 2.1	1	6	0.5	0.526
P-type	54	423.1 ± 4.1	43	424.2 ± 4.6	1	95	0.03	0.859

No. cells indicates the number of cells passing the selection criteria.
